# Assessing the performance of 28 pathogenicity prediction methods on rare single nucleotide variants in coding regions

**DOI:** 10.1186/s12864-025-11787-4

**Published:** 2025-07-07

**Authors:** Jee Yeon Heo, Ju Han Kim

**Affiliations:** 1 Division of Biomedical Informatics, Seoul National University Biomedical Informatics (SNUBI), Seoul National University College of Medicine, Seoul, Korea; 2 Department of Neuropsychiatry, Seoul National University Hospital, Seoul, 03080 Korea

**Keywords:** Nonsynonymous, Single nucleotide variant, Pathogenicity prediction, Variant classification

## Abstract

**Background:**

Accurate pathogenicity prediction of rare variants in coding regions is crucial for prioritizing candidate variants in human diseases and advancing personalized precision medicine. Although many prediction methods have been developed, it remains unclear how they perform specifically on rare variants.

**Results:**

In this study, the performance of 28 pathogenicity prediction methods was assessed using the latest ClinVar dataset, with a focus on rare variants and various allele frequency (AF) ranges. Ten evaluation metrics were employed to comprehensively assess the predictive performance of each method. The methods were selected based on their training approaches, including whether the training dataset was filtered by AF and whether AF was incorporated as a feature. Most methods focused on missense and start-lost variants, covering only a subset of nonsynonymous SNVs. The average missing rate of approximately 10% was observed in these variants, indicating that prediction scores were unavailable for them. MetaRNN and ClinPred, which incorporated conservation, other prediction scores, and AFs as features, demonstrated the highest predictive power on rare variants. For most methods, specificity was lower than sensitivity. Across various AF ranges, most performance metrics tended to decline as AF decreased, with specificity showing a particularly large decline.

**Conclusions:**

These results provide insights into the strengths and limitations of each method in predicting the pathogenicity of rare variants, which may guide future improvements in predictive models. Furthermore, while AF and existing prediction scores offer valuable information for prediction methods, the identification of novel biological features is essential to overcome current limitations and further improve predictive performance.

**Supplementary Information:**

The online version contains supplementary material available at 10.1186/s12864-025-11787-4.

## Background

The development of next-generation sequencing (NGS) has revolutionized our understanding of genetic variations, leading to the discovery of a vast number of genetic variants [1]. These advancements have enabled the identification of a broad spectrum of genetic variations across a wide range of allele frequencies (AFs).

Genetic variation encompasses changes ranging from single nucleotide alterations to large-scale chromosomal rearrangements. Among these, single nucleotide variants (SNVs) are the most prevalent type, accounting for approximately 0.1% of the human genome [2]. Nonsynonymous single nucleotide variants (nsSNVs), which result in amino acid changes in the coding region, are of particular interest because of their potential impact on gene function and their association with various diseases [3].

AF of genetic variants spans a wide spectrum, from common to rare. Rare variants, defined as those with a minor allele frequency (MAF) of less than 0.01 [4], have become a key focus in genetic research owing to their potential contributions to both complex and Mendelian diseases [5, 6].

However, the majority of variants identified by NGS remain of unknown significance. This is because experimental validation of large numbers of variants is often infeasible [7], and association studies require prohibitively large sample sizes to detect rare variants with modest effect sizes and high statistical power [8]. Understanding the functional consequences of rare variants is critical for advancing genetic research, improving disease diagnosis, and refining predictive models in personalized medicine. Various computational prediction methods for pathogenicity classification of variants have been developed and are widely used in many studies to address these challenges.

Although several previous studies have compared the performance of these methods [9–19], how these methods perform specifically on rare variants has not been thoroughly investigated. Therefore, in this study, we assessed the performance of 28 pathogenicity prediction methods, with a focus on rare variants and various AF ranges. This evaluation provides insights into the strengths and limitations of each method in predicting pathogenicity on rare variants, which can guide future improvements in predictive models.

## Methods

Data collection and analysis were performed using Perl and Python. The data and code used for the analysis are available at https://github.com/DNAvigation/Compare.

### Collection of the benchmark dataset

The ClinVar [20] database, which comprises clinically observed genetic variants, was used as the benchmark dataset. To avoid overlap with the training datasets used for the prediction methods, SNVs registered between 2021 and 2023 were selected (*N* = 1,447,467). These SNVs were filtered based on the following criteria. First, SNVs with clinical significance classified as pathogenic, likely pathogenic, or pathogenic/likely pathogenic were labeled as pathogenic, whereas those classified as benign, likely benign, or benign/likely benign were labeled as benign (*N* = 759,388). Second, to reduce misclassification in the curated data, SNVs with a review status of practice_guidelines, reviewed_by_an_expert_panel, or criteria_provided_multiple_submitters_no_conflicts were retained (*N* = 68,685). Third, nsSNVs, including missense, start_lost, stop_gained, and stop_lost variants in coding regions, were selected. After applying all filters, 8,508 nsSNVs remained, comprising 4,891 pathogenic and 3,617 benign variants. These included 5,510 missense, 53 start_lost, 2,940 stop_gained, and 5 stop_lost variants.

### Allele frequency of the benchmark dataset

To select rare variants from the benchmark dataset, six AF datasets from four different databases were collected. The four databases were the Exome Sequencing Project (ESP) [21], the 1000 Genomes Project (1000GP, phase 3) [22], and the Exome Aggregation Consortium (ExAC) [23], and the Genome Aggregation Database (gnomAD, v4.0) [24]. The six AF datasets consisted of the African American samples of ESP (ESP_AA, *N* = 2,217), the European American samples of ESP (ESP_EA, *N* = 4,298), the total samples of 1000GP (1000GP, *N* = 2,504), the total samples of the ExAC (ExAC, *N* = 60,706), the whole genome samples of the gnomAD (gnomAD_G, *N* = 76,215), and the whole exome samples of the gnomAD (gnomAD_E, *N* = 730,947). AF data for ESP, 1000GP, and ExAC were obtained from the dbNSFP database (v4.4a) [25], whereas data for gnomAD were obtained from its database. Rare variants were defined as those with an AF of less than 0.01 in gnomAD. To evaluate performance across various AF ranges, AF was categorized into six intervals, each decreasing by a factor of 10 from 1 to 0.

### Selection of pathogenicity prediction methods

To evaluate the performance of pathogenicity prediction methods on rare variants, precalculated prediction scores from 28 methods were obtained via the dbNSFP.

These methods were categorized into four groups based on their handling of AF in the training dataset, specifically considering whether the dataset was filtered by AF and whether AF was incorporated as a feature.

The first group includes methods specifically trained on rare variants to predict their pathogenicity, such as FATHMM-XF [26], M-CAP [27], MetaRNN [28], MVP [29], REVEL [30], VARITY (ER, R) [31], and gMVP [32]. The second group consists of methods trained using common variants as the benign dataset, including FATHMM-MKL [33], LIST-S2 [34], PrimateAI [35], and VEST4 [36]. The third group comprises methods that incorporate AF as a feature, such as CADD [37, 38], ClinPred [39], DANN [40], Eigen [41], MetaLR [11], and MetaSVM [11]. The final group includes methods that do not utilize AF information, such as DEOGEN2 [42], FATHMM [43], GenoCanyon [44], MutationAssessor [45], MutPred [46], Polyphen2 (HDIV, HVAR) [47], PROVEAN [48], SIFT [49], and SIFT4G [50]. For variants with multiple prediction scores, values corresponding to canonical transcripts were used. Thresholds for distinguishing pathogenic from benign variants were obtained from either the dbNSFP or the original studies.

Because prediction scores were not consistently available for all variants, only 1,154 variants out of the 8,508 in the benchmark dataset, all of which were missense, had prediction scores available from all 28 methods. To avoid significant data loss, all variants with prediction scores for each method were used in the performance comparison.

### Correlation analysis among prediction methods

To investigate the relationships among the 28 prediction methods, a correlation analysis was performed using the Spearman correlation coefficient. First, for methods where a lower score indicates higher risk, such as FATHMM, PROVEAN, SIFT, and SIFT4G, the scores were transformed so that higher scores represent higher risk, aligning with the interpretation of risk across all methods. Next, methods with score ranges outside the 0 to 1, such as CADD, Eigen, FATHMM, MetaSVM, MutationAssessor, and PROVEAN, were scaled before calculating the correlation. Finally, hierarchical clustering was applied to group the methods based on similarities in prediction scores.

### Metrics used for performance evaluation

The ten metrics used to compare the performance of the pathogenic prediction methods include sensitivity, specificity, precision, NPV (negative predictive value), accuracy, F1-score, Matthews correlation coefficient (MCC), geometric mean (G-mean), area under the receiver operating characteristic curve (AUC), and area under the precision-recall curve (AUPRC) [51, 52]. Sensitivity, specificity, precision, NPV, accuracy, F1-score, MCC, and G-mean were calculated based on thresholds from the dbNSFP or the original studies. Unlike other metrics, both AUC and AUPRC are not influenced by the threshold.

Sensitivity (also referred to as the recall or true positive rate) represents the fraction of true positives correctly predicted as positive. Specificity (also referred to as the true negative rate) represents the fraction of true negatives correctly predicted as negative. Precision (also referred to as positive predictive value) represents the fraction of true positives among all the predicted positives. Negative predictive value (NPV) represents the fraction of true negatives among all the predicted negatives. Accuracy represents the fraction of correct predictions (true positives and true negatives) out of all predictions. The F1-score represents the harmonic mean of precision and sensitivity, ranging from 0 to 1, with a higher score indicating better performance. MCC represents the correlation coefficient between the observed and predicted classifications. It ranges from − 1 to 1, where 1 indicates a perfect prediction, 0 indicates a prediction that is no better than random, and − 1 indicates a completely incorrect prediction. G-mean represents the balance between sensitivity and specificity, and is particularly useful for evaluating highly imbalanced datasets.

The receiver operating characteristic (ROC) curve illustrates the trade-off between sensitivity and specificity at different thresholds, whereas the precision-recall (PR) curve shows the trade-off between precision and recall across various thresholds. The AUC and AUPRC provide an overall measure of classification performance across all possible thresholds. A value of 1 for the AUC and AUPRC indicates perfect classification, whereas a value of 0.5 suggests performance equivalent to random chance. The best model was selected based on having the highest AUC. It can be calculated using the following formula:

$$\:\text{S}\text{e}\text{n}\text{s}\text{i}\text{t}\text{i}\text{v}\text{i}\text{t}\text{y}=\:\frac{\text{T}\text{P}}{\text{T}\text{P}+\text{F}\text{N}}$$

$$\:\text{S}\text{p}\text{e}\text{c}\text{i}\text{f}\text{i}\text{c}\text{i}\text{t}\text{y}=\:\frac{\text{T}\text{N}}{\text{T}\text{N}+\text{F}\text{P}}$$

$$\:\text{P}\text{r}\text{e}\text{c}\text{i}\text{s}\text{i}\text{o}\text{n}=\:\frac{\text{T}\text{P}}{\text{T}\text{P}+\text{F}\text{P}}$$

$$\:\text{N}\text{P}\text{V}=\:\frac{\text{T}\text{N}}{\text{T}\text{N}+\text{F}\text{N}}$$

$$\:\text{A}\text{c}\text{c}\text{u}\text{r}\text{a}\text{c}\text{y}=\:\frac{\text{T}\text{P}+\text{T}\text{N}}{\text{T}\text{P}\:+\text{T}\text{N}+\text{F}\text{P}+\text{F}\text{N}}$$

$$\:\text{F}1\:\text{s}\text{c}\text{o}\text{r}\text{e}=2\times\:\frac{\text{P}\text{r}\text{e}\text{c}\text{i}\text{s}\text{i}\text{o}\text{n}\:\times\:\text{S}\text{e}\text{n}\text{s}\text{i}\text{t}\text{i}\text{v}\text{i}\text{t}\text{y}}{\text{P}\text{r}\text{e}\text{c}\text{i}\text{s}\text{i}\text{o}\text{n}+\text{S}\text{e}\text{n}\text{s}\text{i}\text{t}\text{i}\text{v}\text{i}\text{t}\text{y}}$$

$$\:\text{M}\text{C}\text{C}=\:\frac{\left(\text{T}\text{P}\times\:\text{T}\text{N}\right)-\left(\text{F}\text{P}\times\:\text{F}\text{N}\right)}{\sqrt{\left(\text{T}\text{P}+\text{F}\text{P}\right)\left(\text{T}\text{P}+\text{F}\text{N}\right)\left(\text{T}\text{N}+\text{F}\text{P}\right)\left(\text{T}\text{N}+\text{F}\text{N}\right)}}$$

$$\:\text{G}-\text{M}\text{e}\text{a}\text{n}=\:\sqrt{\text{S}\text{e}\text{n}\text{s}\text{i}\text{t}\text{i}\text{v}\text{i}\text{t}\text{y}\times\:\text{S}\text{p}\text{e}\text{c}\text{i}\text{f}\text{i}\text{c}\text{i}\text{t}\text{y}}$$

where TP, FP, TN, and FN represent true positive, false positive, true negative, and false negative, respectively.

## Results

### Summary of pathogenicity prediction methods

**Fig. 1:**
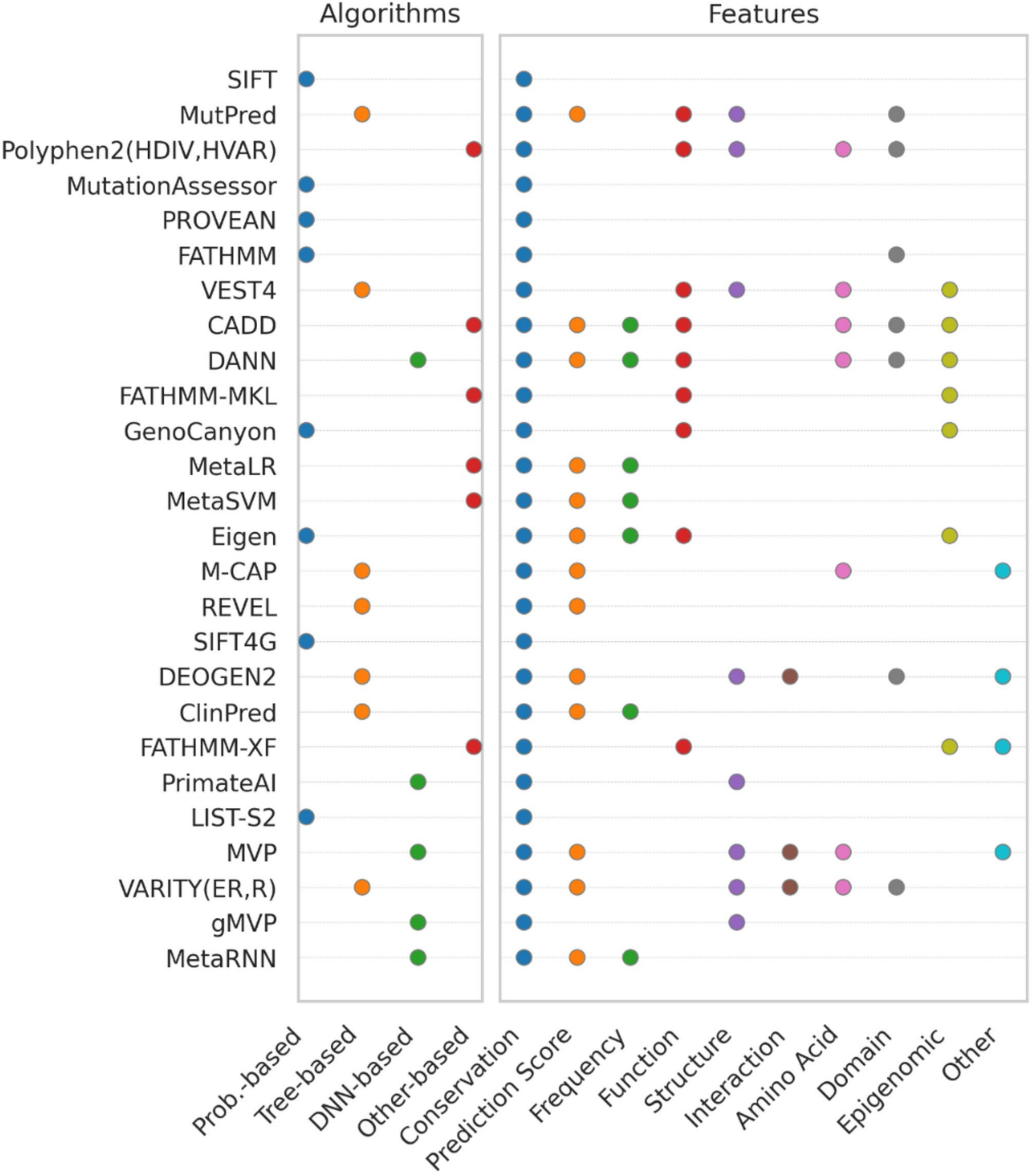
Summary of algorithms and features used in 28 pathogenicity prediction methods. Based on the algorithms used, each method was labeled as Probabilistic-based, Tree-based, DNN-based, and Other-based. Based on the features included, each method was labeled as Conservation, Prediction Score, Frequency, Function, Structure, Interaction, Amino Acid, Domain, and Other-property

The characteristics of the 28 pathogenicity prediction methods assessed in this study are summarized in Table S1. Figure 1 shows the categories of algorithms and features used in these methods.

These methods employed a range of approaches, from probabilistic-based and traditional machine learning to deep learning, along with various biological properties as features to build a model. Early prediction methods, such as SIFT and PolyPhen2, were developed using protein sequences and probabilistic-based algorithms, including position-specific scoring matrices (PSSMs), hidden Markov models (HMMs), expectation-maximization (EM), combinatorial entropy formalism (CEF), and Bayes’ rule. With the increase in publicly available variant data, prediction methods were developed based on traditional machine-learning algorithms, such as random forest (RF) and support vector machine (SVM). More recently, deep-learning-based methods emerged. Among the tree-based prediction methods, random forest (RF), gradient boosting tree (GBT), and eXtreme gradient boosting (XGBoost) were commonly used. These algorithms primarily employ ensemble classifiers, which train multiple weak classifiers, such as decision trees, and combine their outputs (e.g., voting) to achieve better predictive performance. In deep neural network (DNN)-based methods, architectures such as DNN, ResNet, graph attention network (GAT), and recurrent neural network (RNN) were used. In other-based methods, logistic regression (LR), multiple kernel learning (MKL), naïve bayes (NB), and support vector machine (SVM) were employed.

The most commonly used feature is conservation, which was utilized in all methods. These features include sequence homology using multiple sequence alignment and evolutionary conservation metrics such as phastCons, phyloP, and GERP. The second most frequently used feature is other prediction scores, such as those from methods such as SIFT and PolyPhen2. Among these, SIFT was the most frequently used prediction score, appearing in 13 prediction methods, such as CADD, ClinPred, DANN, Eigen, M-CAP, MetaLR, MetaSVM, MetaRNN, MutPred, MVP, REVEL, and VARITY (R, ER) (Fig. S1). The metaRNN incorporates the most prediction scores from 16 different prediction methods, including CADD, DEOGEN2, Eigen, FATHMM-XF, GenoCanyon, M-CAP, MutationAssessor, MutPred, MVP, PolyPhen2 (HDIV, HVAR), PrimateAI, PROVEAN, REVEL, SIFT, and VEST4.

Frequency properties, such as AF from ESP, 1000GP, ExAC, and gnomAD, were used as features in seven prediction methods. AF was also used as a criterion for filtering rare variants or selecting common variants as the benign dataset in methods such as FATHMM-MKL, FATHMM-XF, gMVP, LIST-S2, M-CAP, MetaRNN, MVP, PrimateAI, REVEL, VARITY (ER, R), and VEST4. Functional properties, including DNA-binding sites and CpG island locations, were incorporated into nine methods. Structural properties, such as secondary structure, solvent accessibility, transmembrane helices, and coiled-coil structures, were used in nine methods. Three methods used features related to interaction properties, such as protein-protein interactions. Seven methods considered amino acid properties, such as polarity, charge, and substitution matrices like BLOSUM62 and PAM250. Additionally, seven methods incorporated domain-related properties, such as those from Pfam, whereas seven others have utilized epigenetic features, including methylation sites and histone modifications, especially for pathogenic prediction in noncoding regions. Finally, other properties, such as pathway and gene tolerance metrics such as the gene damage index (GDI), residual variance intolerance score (RVIS), and probability of being loss-of-function intolerant (pLI), were used in four methods. Most of these methods were designed to distinguish pathogenic from benign variants in coding regions, whereas seven methods, such as CADD, DANN, Eigen, FATHMM-MKL, FATHMM-XF, GenoCanyon, and VEST4, which incorporate epigenomic properties, have been developed to predict pathogenic variants in both coding and noncoding regions.

### Variant types and missing rates in prediction methods

**Fig. 2:**
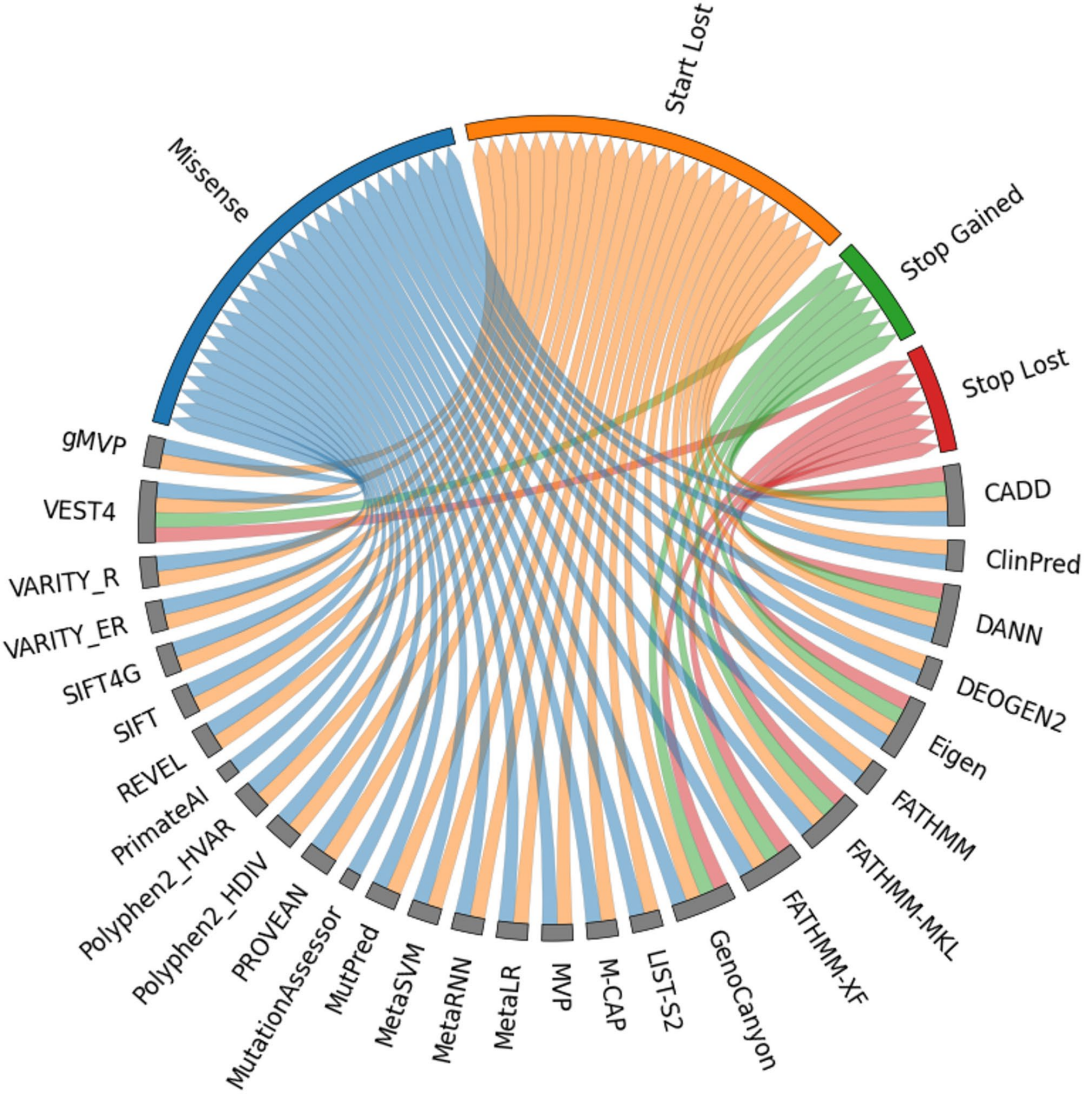
Coverage of variant types in 28 prediction methods. The chord diagram illustrates the relationships between variant types and prediction methods, represented as curved arcs within a circle. Gray boxes denote prediction methods, while the blue, orange, green, and red boxes represent missense, start_lost, stop_gained, and stop_lost variants, respectively. The colored arcs connect the variant types covered by each prediction method

The benchmark dataset consisted of missense (*N* = 5,510, 64.76%), start_lost (*N* = 53, 0.62%), stop_gained (*N* = 2,940, 34.56%), and stop_lost (*N* = 5, 0.06%) variants. Figure 2 illustrates the coverage of variant type for each method in this dataset. Most methods focused on missense and start_lost variants, covering only two of the four variant types of nsSNVs, while MutationAssessor and PrimateAI covered only missense variants. Seven prediction methods, including CADD, DANN, Eigen, FATHMM-MKL, FATHMM-XF, GenoCanyon, and VEST4, covered all types of nsSNVs. These methods were developed for pathogenic variant prediction in both coding and noncoding regions.

**Fig. 3:**
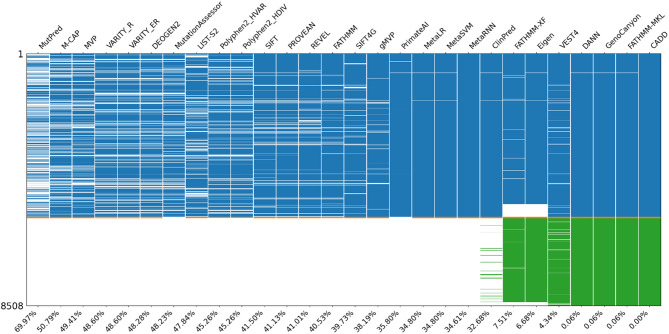
Overall missing rate for each method in the benchmark dataset (*N* = 8,508). Each column represents a method, and each row corresponds to a variant. The blue, orange, green, and red regions correspond to the variant types of missense (*N* = 5,510), start_lost (*N* = 53), stop_gained (*N* = 2,940), and stop_lost (*N* = 5), respectively. The numbers below indicate the overall missing rate for each method

Figure 3 shows the missing data for each method in the benchmark dataset (*N* = 8,508). Each column represents a prediction method, and each row corresponds to a variant, which has been grouped by variant type and sorted by chromosome and position. The colored regions represent the prediction scores available for variants, while the white regions indicate the absence of scores. Because most prediction methods did not cover stop-gained variants, which account for a large portion of this dataset, the overall missing rate exceeds 30%. The missing rates and the number of available variants grouped by variant type for each method are listed in Table S2. Even when only missense variants were considered, most methods had a missing rate of approximately 10%, with MutPred having the highest missing rate of 54.52%, which is similar to previous research [53]. ClinPred covered some stop-gained variants, but its coverage was less than 7%. Additionally, FATHMM-XF and Eigen did not provide prediction scores for chromosome X.

### Correlation between prediction methods

**Fig. 4:**
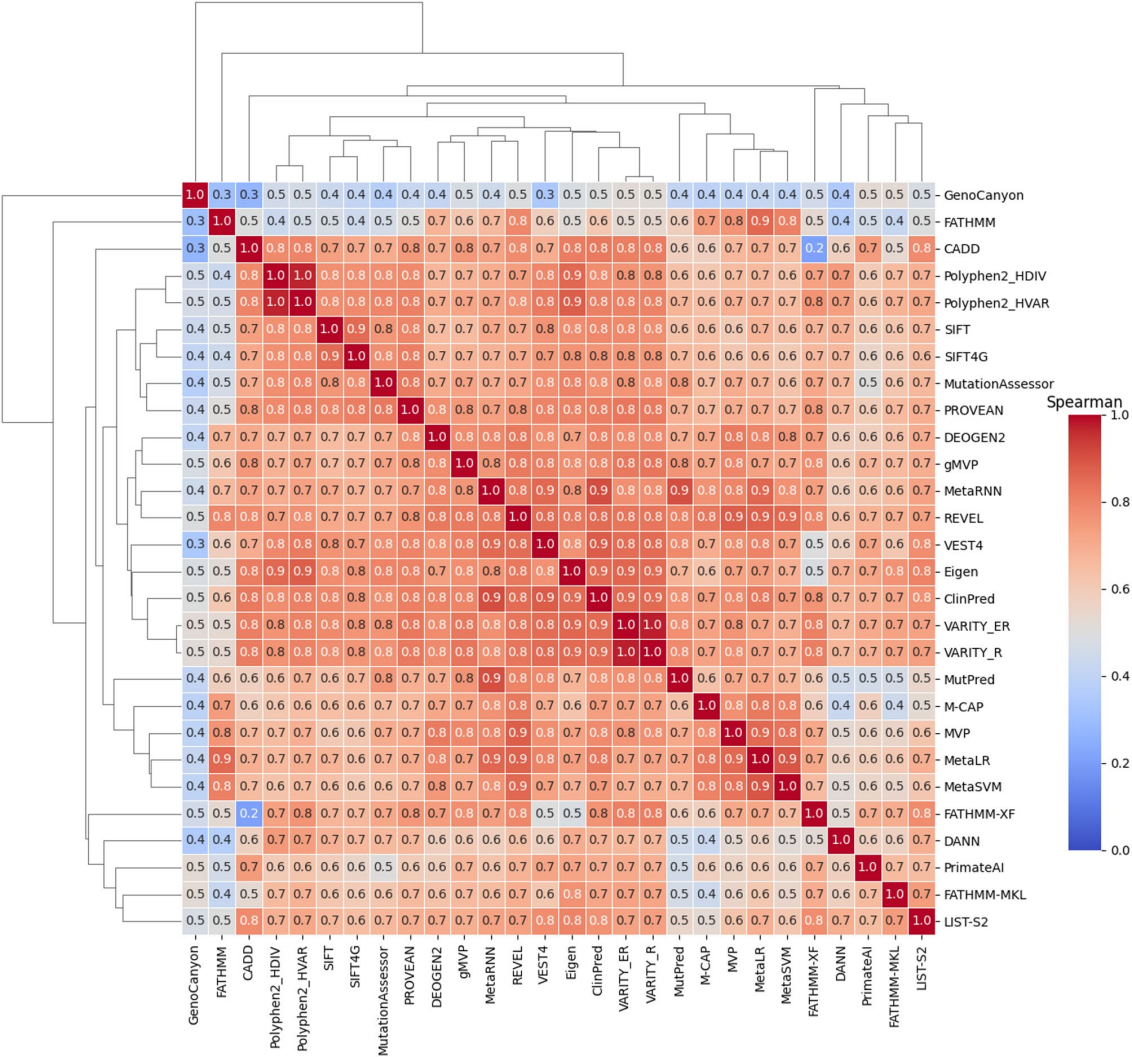
Correlation of prediction scores among 28 prediction methods. The heatmap displays the Spearman rank correlation coefficients between prediction methods, where colors closer to red indicate stronger positive correlations. Hierarchical clustering reveals the relationships and similarities among the methods (*N* = 8,508)

To quantify the similarities among the prediction scores of the 28 methods, the Spearman rank correlation coefficient was calculated using all variants (*N* = 8,508). The heatmap shows that the methods exhibit positive correlations (Fig. 4).

GenoCanyon was weakly to moderately correlated with other methods, while FATHMM, M-CAP, and MutPred were weakly to moderately correlated with only a subset of methods. The other methods were moderately to highly correlated with each other. The lowest correlation was between CADD and FATHMM-XF, whereas ClinPred was highly correlated with most prediction methods. In hierarchical clustering, methods derived from the same study, such as VARITY_R and VARITY_ER, PolyPhen2_HDIV and PolyPhen2_HVAR, SIFT and SIFT4G, and MetaLR and MetaSVM, were clustered together. Additionally, the Spearman rank correlation coefficient was calculated to quantify similarities among binary classifications (Fig. S2). The correlation obtained using binary classification based on the threshold of each method was lower than that obtained using prediction scores, indicating that thresholds for each prediction method could lead to varying classification results. CADD, GenoCanyon, and PrimateAI were the least correlated with the other methods, whereas MetaRNN, ClinPred, REVEL, and VARITY (ER, R) were highly correlated with each other.

### Distribution of the benchmark dataset across different allele frequency ranges

To investigate the distribution of rare variants in the benchmark dataset, six AF datasets from four databases were used, including ESP (AA and EA), 1000GP, ExAC, and gnomAD (exome and genome), which are widely used as features in prediction methods or as filtering criteria for training datasets. Missense and start_lost variants in the benchmark dataset (*N* = 5,563), found in most prediction methods, showed that pathogenic variants were predominantly distributed with AF < 1e-03, whereas benign variants were spread across a wide range of AFs, including both common and rare variants (Fig. S3).

ESP_AA, ESP_EA, and 1000GP were measured up to an AF of 1e-04, while ExAC and gnomAD_G were measured down to an AF of 1e-06, and gnomAD_E was measured down to an AF of 1e-07. In ESP (AA, EA) and 1000GP, where the sample size is fewer than 5,000, AF was measured only up to 1e-04, and AF < 1e-06 represents AF not observed in the corresponding database. However, in ExAC and gnomAD (G, E), with a larger number of samples, AFs lower than 1e-04 were measured. The stop_gained and stop_lost variants (*N* = 2,945) were mostly pathogenic, and a similar distribution was observed for the missense and start_lost variants (Fig. S4).

The parallel categories diagram illustrates the flow of AF changes across six AF ranges (Fig. S5). Some variants exhibited changes in AF across these ranges. Most benign variants that were not observed in AFs in ESP (AA, EA) and 1000GP were observed in ExAC and gnomAD (G, E) with larger sample sizes, whereas for pathogenic variants, AFs remained absent.

### Performance comparison of prediction methods on rare variants

The performance of 28 prediction methods was assessed using the rare missense and start_lost variants, which consisted of 1,951 pathogenic and 2,638 benign variants (*N* = 4,589). Rare variants were selected based on an AF of less than 0.01 in gnomAD_E.

The distribution of the prediction scores for each method clearly exhibited a bimodal pattern in ClinPred, MetaLR, MetaRNN, MetaSVM, REVEL, and VARITY (ER, R), whereas the other methods did not (Fig. S6).

**Fig. 5:**
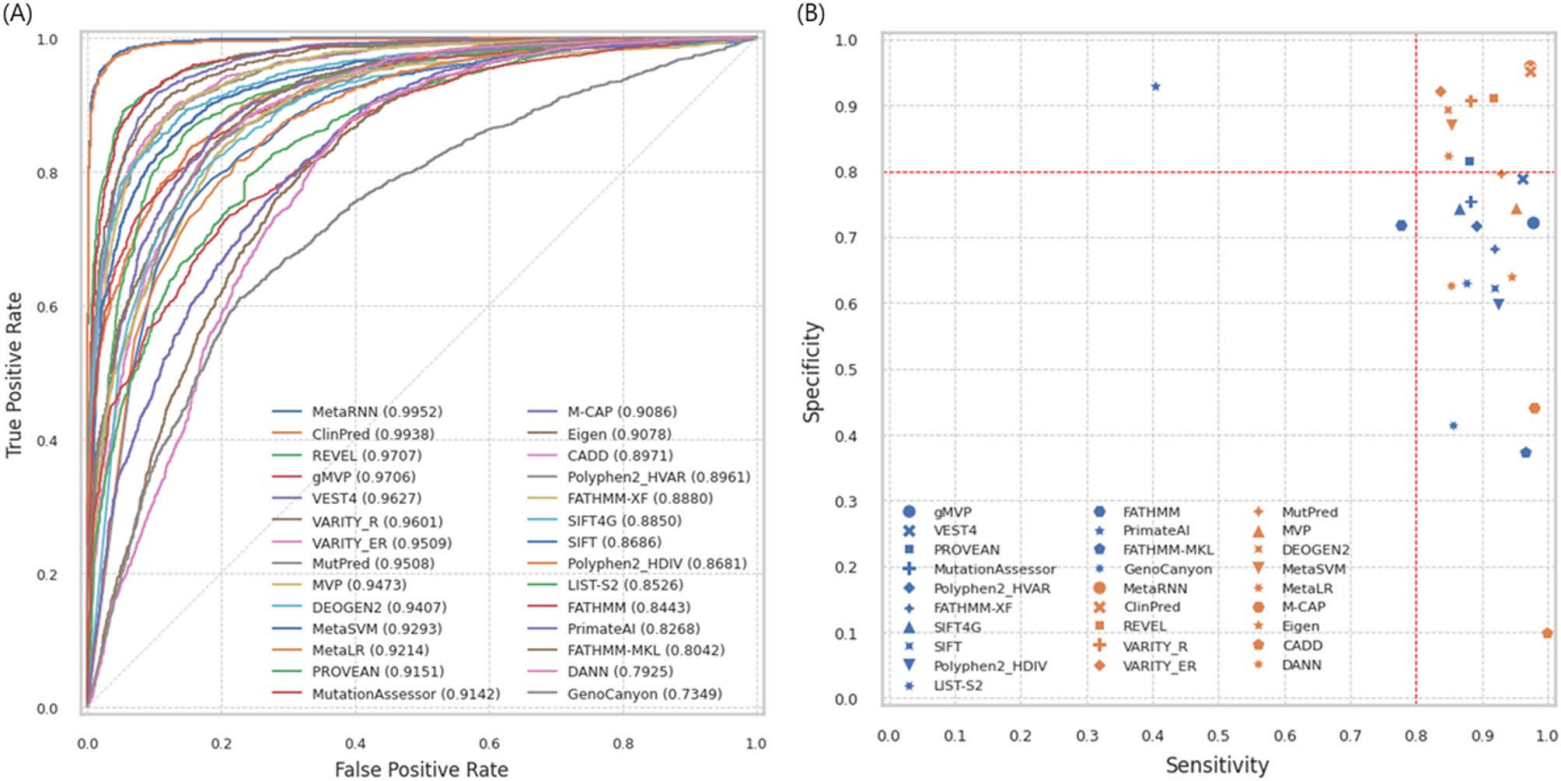
Performance comparison of 28 prediction methods on rare variants (*N* = 4,589). **A** The ROC curve shows the performance comparison of 28 prediction methods. **B** The sensitivity and specificity plot illustrates the relationship between sensitivity and specificity. Higher sensitivity and specificity indicate better performance. Fourteen blue markers represent methods without other pathogenic prediction scores as features, while fourteen orange markers represent methods that incorporate them.

A summary of the ten metrics evaluated for each method is provided in Table S3. The ROC curves were plotted, and the AUC was calculated (Fig. 5A). The results varied substantially across the 28 prediction methods, with AUCs ranging from 0.7349 to 0.9952 and AUPRCs ranging from 0.6517 to 0.9938. The best-performing methods, MetaRNN (AUC = 0.9952) and ClinPred (AUC = 0.9938), which integrated other prediction scores and AF as features, outperformed others in distinguishing pathogenic from benign variants.

To evaluate the performance of binary classification, eight metrics were calculated for the threshold recommended by the authors, namely, sensitivity, specificity, precision, NPV, accuracy, F1-score, MCC, and the G-mean. The sensitivity ranged from 0.4052 to 0.9995 (median = 0.9047), and eight methods (CADD, ClinPred, FATHMM-MKL, M-CAP, MVP, MetaRNN, VEST4, and gMVP) had a sensitivity > 0.95. The specificity ranged from 0.0982 to 0.9587 (median = 0.7435), and two methods (ClinPred, MetaRNN) had a specificity > 0.95. The specificity of most methods was much lower than the sensitivity. The sensitivity and specificity plot shows that most prediction methods tend to overestimate the number of pathogenic variants, leading to high sensitivity but low specificity (Fig. 5B), which aligns with previous research [9, 10]. In particular, methods that integrated multiple pathogenic prediction scores as features had a lower tendency to overestimate sensitivity than methods that did not use them, which had a specificity below 0.8. The greater the imbalance between sensitivity and specificity is, the larger the performance difference in terms of the precision and NPV (Fig. S8B). The precision ranged from 0.4505 to 0.9457 (median = 0.7250), and the NPV ranged from 0.6830 to 0.9962 (median = 0.9036). The accuracy ranged from 0.4814 to 0.9649 (median = 0.7947). Only two methods, MetaRNN (0.9649) and ClinPred (0.9607), had accuracies > 0.95. MetaRNN and ClinPred consistently showed the highest F1-scores, MCCs, and G-means. The performance of the prediction methods on rare variants was slightly lower than that on all variants, which also included AF ≥ 1% (Fig. S7, Fig. S8A, Table S4). The order of the AUC between rare and all variants remained largely consistent, except for Eigen.

### Performance comparison of prediction methods across various AF ranges

To investigate why the performance is lower on rare variants than on all variants, performance was evaluated across various AF ranges. The AF ranges were categorized based on AFs from gnomAD_E, and the performance of the prediction methods was assessed across six AF ranges using missense and start_lost variants. All prediction methods classified variants into pathogenic and benign using the threshold that was recommended by the author, regardless of the AF range.

**Fig. 6:**
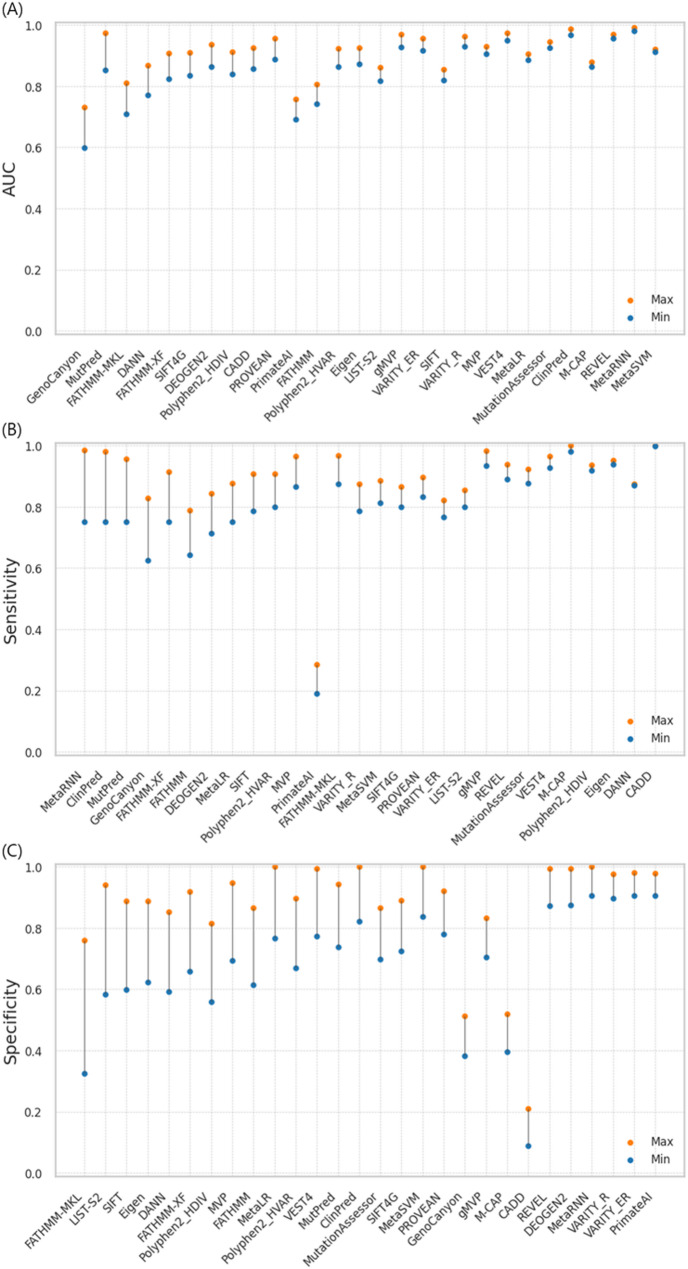
Difference between the minimum and maximum performance across AF ranges. Each vertical line represents the difference between the maximum and minimum performance, sorted in descending order. **A** AUC, **B** Sensitivity, **C** Specificity

Most methods exhibited varying performance across AF ranges and showed differences between the highest and lowest performance within these ranges (Table S5). The difference between the minimum and maximum AUCs across AF ranges for the prediction methods ranged from 0.0087 to 0.1308 (median = 0.0482) (Fig. 6A). The smallest differences were observed for MetaSVM (0.0087), MetaRNN (0.0106), REVEL (0.0145), M-CAP (0.016), ClinPred (0.0196), and MutationAssessor (0.0198), whereas the largest difference was observed for GenoCanyon (0.1308). The sensitivity difference across these ranges varied from 0.0017 to 0.2343 (median = 0.0803) (Fig. 6B). The smallest differences were observed for CADD (0.0017), DANN (0.0039), Eigen (0.0145), Polyphen2_HDIV (0.0163), and M-CAP (0.0187), whereas the largest difference was observed for MetaRNN (0.2343). The difference in specificity varied from 0.0737 to 0.4333 (median = 0.1723) (Fig. 6C). The smallest differences were observed for PrimateAI (0.0737), VARITY_ER (0.0744), VARITY_R (0.0781), and MetaRNN (0.0944), while the largest difference was observed for FATHMM-MKL (0.4333).

**Fig. 7:**
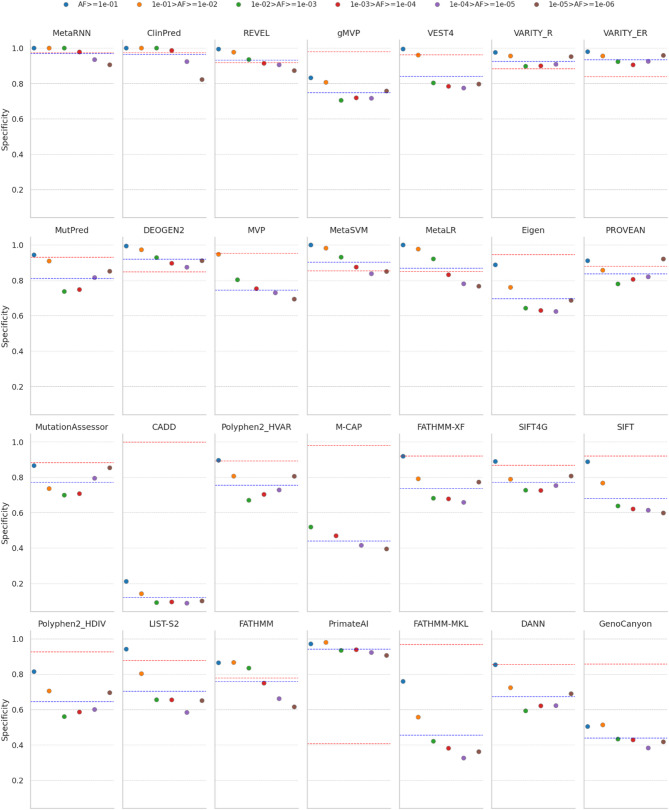
Specificity performance of 28 prediction methods across six AF ranges. Specificity tends to decline with decreasing AF. The red horizontal line represents sensitivity, while the blue horizontal line represents specificity on all missense and start_lost variants (*N* = 5,563). The methods are ordered in descending order of AUC

For benign variants, where data were available across six AF ranges, the specificity tended to be lower for variants with lower AFs in most methods (Fig. 7). This trend was also observed in common variants (1% ≤ AF < 25%) in a previous study [10].

Specificity tended to decline with decreasing AF in methods whose training datasets were filtered by AF or that incorporated AF as a feature, whereas those that do not utilize AF information did not exhibit this trend. For the seven methods, ClinPred, MetaRNN, MetaLR, MetaSVM, CADD, DANN, and Eigen, which incorporated AFs as features, specificity tended to decline as the AF decreased. MetaRNN, which was trained by filtering the dataset with AFs < 1%, showed a smaller decline in specificity compared to ClinPred, which was trained without AF filtering. However, its specificity also decreased for variants with AF < 0.1%. Methods such as LIST-S2, FATHMM-MKL, and VEST4, which were trained using common variants as benign, exhibited decreased specificity for both AF > 1% and AF ≤ 1%. VARITY (ER, R) and gMVP were trained by filtering out extremely rare benign variants with AF < 0.1%. The performance of these methods decreased for common variants but remained stable for rare variants as the AF decreased. However, their overall performance remained poor. Probabilistic-based methods that did not utilize AF information exhibited a U-shaped pattern, where specificity decreased and then increased as AF decreased. This trend was observed in methods such as MutationAssessor, PolyPhen-2 (HDIV, HVAR), PROVEAN, and SIFT4G. In the case of PrimateAI, which only used common variants as benign in humans and other primates and did not include pathogenic variants, specificity was higher than sensitivity, and performance remained relatively stable despite the decrease in AF.

In contrast, for pathogenic variants, data were available for an AF < 0.1%, which were categorized into three ranges. Sensitivity tended to increase as the AF decreased, supporting the general tendency that variants with lower AF are more likely to be pathogenic (Fig. S9).

### Performance of the prediction methods in stop_gained and stop_lost variants

The performance of the seven prediction methods was assessed using stop_gained and stop_lost variants, comprising 2,940 pathogenic and 5 benign variants (*N* = 2,945). In this dataset, all pathogenic variants were rare variants, with an AF < 0.1%, and more than 50% were not observed in the gnomAD_E. Among the five benign variants, one was a common variant with an AF > 0.1, while four were rare variants, with AFs ranging from 1e-5 to 1e-4. The performance metrics are summarized in Table S6.

The MCC and G-mean, which are appropriate for evaluating performance on highly imbalanced datasets, ranged from − 0.0159 to 0.1038 and 0 to 0.9269, respectively. Four methods (CADD, DANN, Eigen, and FATHMM-MKL) showed sensitivity ranging from 0.8431 to 1, while specificity was 0. This resulted in the G-mean and MCC of 0 or even negative, suggesting performance similar to random guessing. These methods tended to overestimate the number of pathogenic variants, leading to the misclassification of benign variants as pathogenic and exhibiting low specificity, which results in a high false positive rate. The small sample size of the benign variants (*N* = 5) and the tendency to overestimate pathogenicity may have contributed to the specificity being 0.

In contrast, three methods (FATHMM-XF, GenoCanyon, and VEST4) showed sensitivity ranging from 0.0622 to 0.8592, while specificity ranged from 0.75 to 1. These methods performed contrary to the trend observed in missense and start-lost variant datasets, overestimating the number of benign variants and leading to poor identification of pathogenic variants due to high false negative rates, particularly in FATHMM-XF.

## Discussion

Computational pathogenicity prediction methods have been widely used to distinguish pathogenic from benign variants. Selecting the appropriate methods is crucial for prioritizing candidate variants in human disease. In this study, the performance of 28 pathogenicity prediction methods was evaluated using ten metrics, focusing on rare variants and various AF ranges, while examining the characteristics of these methods.

Most prediction methods were trained on known pathogenic and benign variants, which are sourced from public databases. However, using overlapping variants in both training and evaluation can result in inflated performance metrics [12]. To minimize this bias and ensure a fair evaluation, the study employed methods published until 2022 and used ClinVar variants submitted after 2021 as the benchmark dataset. This approach was designed to prevent overlap with training data and support an unbiased performance assessment.

The nonsynonymous SNVs (nsSNVs), which alter amino acids in coding regions, include missense, start-lost, stop-gain, and stop-lost variants. However, most prediction methods focused only on the missense and start_lost variants while excluding stop_gained and stop_lost variants. Methods based on sequence homology using protein sequences, such as SIFT, PolyPhen2, MutationAssessor, PrimateAI, gMVP, and others, may be limited in evaluating variants like stop-gain or stop-lost, since stop codons are not represented in amino acid sequences. Additionally, using prediction scores from methods that were developed to assess only certain variant types as features may limit the applicability of the model, since prediction scores for other variant types are unavailable. The missing rate represents the proportion of variants for which prediction scores are unavailable. Because most prediction methods did not cover stop codon related variants, which represent a large portion of this dataset, the overall missing rate was high. Even when only missense variants were considered, most methods had a missing rate of approximately 10%. These missing rates are due primarily to differences in the annotation information of proteins or transcripts referenced by each method and by whether the features required for each method were available for a given variant.

In the evaluation of rare variants, MetaRNN and ClinPred achieved the highest discriminative power across all performances. These methods incorporated only conservations, other prediction scores, and AFs as features. MetaRNN employed a deep learning algorithm, specifically a recurrent neural network (RNN). However, methods such as gMVP and PrimateAI, which also utilized deep learning-based algorithms, demonstrated poorer performance. This suggests that the effectiveness of deep learning approaches may vary depending on the model architecture and the types of features in the training data. ClinPred employed a random forest (RF), which is a tree-based algorithm. Additionally, methods using tree-based algorithms, such as MutPred, REVEL, VARITY (ER, R), and VEST4, also demonstrated relatively good performance. Additionally, methods that trained the dataset by filtering for rare variants or using AF as features generally performed well. Probabilistic-based methods, such as SIFT and PolyPhen2, which were commonly used as features in other methods, generally showed poor performance, both on the rare variant dataset and on the entire dataset.

Most prediction methods tended to exhibit higher sensitivities than specificities, suggesting that some predicted pathogenic variants are actually benign. This discrepancy was more pronounced in methods that did not incorporate other prediction scores as features. While other prediction scores are useful features for improving performance, they seem to have limitations in expanding to different variant types and addressing the missing rate. Therefore, it is necessary to discover diverse biological features that can be used instead of prediction scores to enhance generalizability.

Most methods showed performance differences across AF ranges, particularly in specificity. Specificity tended to decline with decreasing AF in methods trained on AF-filtered datasets or those that incorporated AF as a feature. In contrast, probabilistic-based methods that did not use AF information exhibited a U-shaped pattern, with specificity decreasing and then increasing as AF decreased. And these methods generally showed lower overall performance compared to models that incorporated AF information.

In real datasets, such as the benchmark dataset, pathogenic variants are predominantly concentrated in the rare AF range near zero, and benign variants are distributed not only near an AF of 1 but also across various AF ranges. Therefore, relying only on common variants as benign may lead to biased predictions and reduced specificity. Additionally, an imbalance between rare benign and pathogenic variants in the training dataset may contribute to the reduced specificity observed in the low-AF ranges. Rather than training by filtering variants based on AF, it may be more beneficial to include variants that allow for a balanced distribution of both pathogenic and benign variants within each AF range. Such an approach can better capture the real-world distribution of variants and enhance the robustness of predictors across various AF ranges. And proxy-labeled benign datasets generated by filtering large population databases such as ExAC and gnomAD based on AF thresholds may contain noise, as these variants are not confirmed to be truly benign. Methods such as M-CAP, FATHMM-XF, and REVEL, which used filtering with AF < 1% to select benign variants, may have included potentially pathogenic variants, thereby introducing noise that can reduce specificity. Therefore, using curated datasets with clinically validated labels for training may help reduce noise and potentially improve the reliability of model performance. Adjusting thresholds depending on data conditions for specific AF ranges has the potential to help maintain sensitivity while mitigating the loss of specificity.

As rare variants in coding regions have been increasingly discovered and recognized for their clinical significance, various pathogenicity prediction methods were developed. However, there remains a need for improved methods to enhance pathogenicity prediction and facilitate the identification of disease-associated variants. These results provide insights into the strengths and limitations of each method in predicting the pathogenicity of rare variants, which can guide future improvements in predictive models. Furthermore, while AF and existing prediction scores offer valuable information for prediction methods, the identification of novel biological features is essential to overcome current limitations and further improve predictive performance.

## Conclusions

With the advancement of NGS technology, many SNVs have been discovered, leading to the development of various methods for distinguishing pathogenic from benign variants. However, the performance evaluation of these methods on rare variants has not yet been conducted. This study evaluated the performance of 28 pathogenicity prediction methods on rare variants of coding regions and various AF ranges across ten metrics. Most prediction methods covered the missense and start_lost variants of nsSNVs and had missing prediction scores. MetaRNN and ClinPred, which incorporated conservation, other prediction scores, and AFs as features, demonstrated the highest predictive power on rare variants and under various AF conditions. For most methods, the specificity tended to be lower than the sensitivity, and performance metrics decreased as AF decreased, with specificity being particularly affected. Overestimated sensitivity can lead to an increased number of false positives, raising reliability concerns in clinical applications. These findings provide insights into the strengths and limitations of each method in predicting the pathogenicity of rare variants, which can guide future improvements in predictive models.

## Supplementary Information

Supplementary Material 1.

Supplementary Material 2.

## Data Availability

The data for this study can be found below. ClinVar(clinvar_20201226.vcf, clinvar_20231230.vcf) https://www.ncbi.nlm.nih.gov/clinvar/ dbNSFP(v4.4a) https://sites.google.com/site/jpopgen/dbNSFP gnomAD(v4) https://gnomad.broadinstitute.org/data The benchmark dataset and code have been uploaded to the GitHub repository. https://github.com/DNAvigation/Compare.

## Abbreviations

1000GP: 1000 genomes project
AF: Allele frequency
AUROC: Receiver operating characteristic curve
CEF: Combinatorial entropy formalism
DNN: Deep neural network
EM: Expectation-maximization
ESP: Exome sequencing project
ExAC: Exome aggregation consortium
GAT: Graph attention network
GBT: Gradient boosting tree
GDI: Gene damage index
G-mean: Geometric mean
gnomAD: Genome aggregation database
HMM: Hidden markov model
LR: logistic regression
MAF: Minor allele frequency
MCC: Matthews correlation coefficient
MKL: Multiple kernel learning
NB: Naive bayes
NGS: next-generation sequencing
NPV: Negative predictive value
nsSNV: Nonsynonymous SNV
pLI: probability of being loss of function intolerant
PSSM: position-specific scoring matrics
RF: Random forest
RNN: Recurrent neural network
ROC: Area under receiver operating characteristic curve
RVIS: Residual variance intolerance score
SNV: Single nucleotide variant
SVM: Support vector machine
XGBoost: eXtreme gradient boosting
