# Supplementary material for: Assessing the performance of 28 pathogenicity prediction methods on rare single nucleotide variants in coding regions
Source: BMC Genomics. 2025 Jul 7;26:641. doi: 10.1186/s12864-025-11787-4 (PMC12235850; doi:10.1186/s12864-025-11787-4)
Supplement: Supplementary file 2 — Supplementary Material 2. [file 12864_2025_11787_MOESM2_ESM.docx]

**Assessing the performance of 28 pathogenicity prediction methods on rare single-nucleotide variants in coding regions**

Jee Yeon Heo,^1^ and Ju Han Kim^*,1,2^

^1^Seoul National University Biomedical Informatics (SNUBI), Division of Biomedical Informatics, Seoul National University College of Medicine, Seoul, 03080, Korea

^2^Department of Neuropsychiatry, Seoul National University Hospital, Seoul, 03080, Korea

^*^Correspondence: juhan@snu.ac.kr


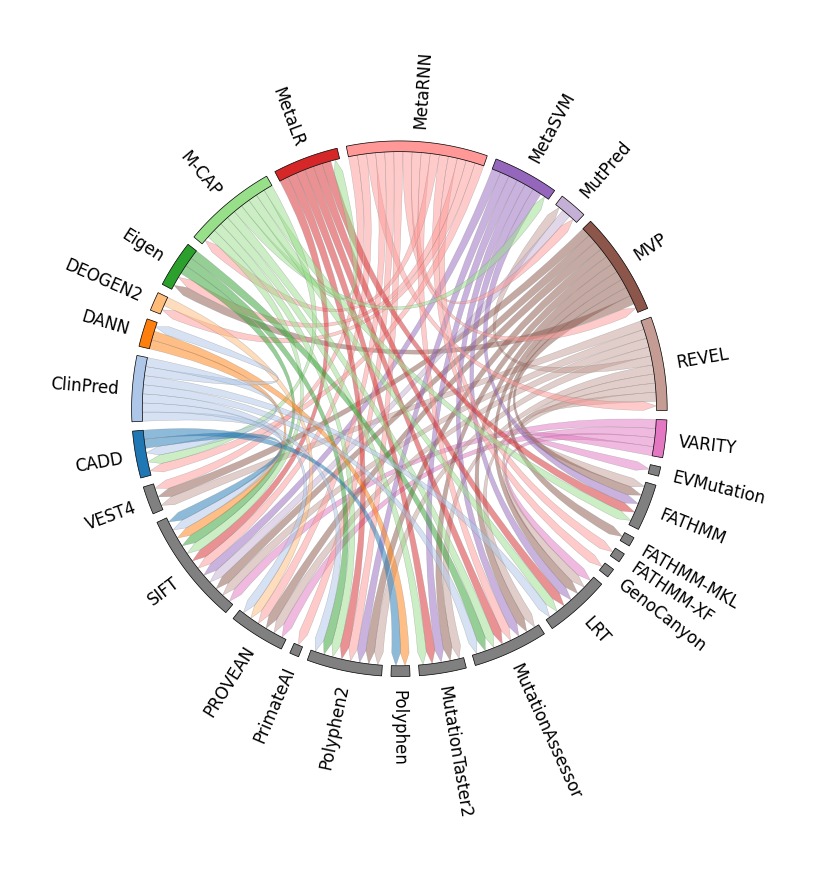


Fig. S1 Prediction methods using other prediction scores as features.

The chord diagram illustrates the relationship among prediction methods, represented as curved arcs within a circle. Gray boxes indicate prediction methods that were used as features, while the other colored boxes represent prediction methods that utilized other prediction scores as features.


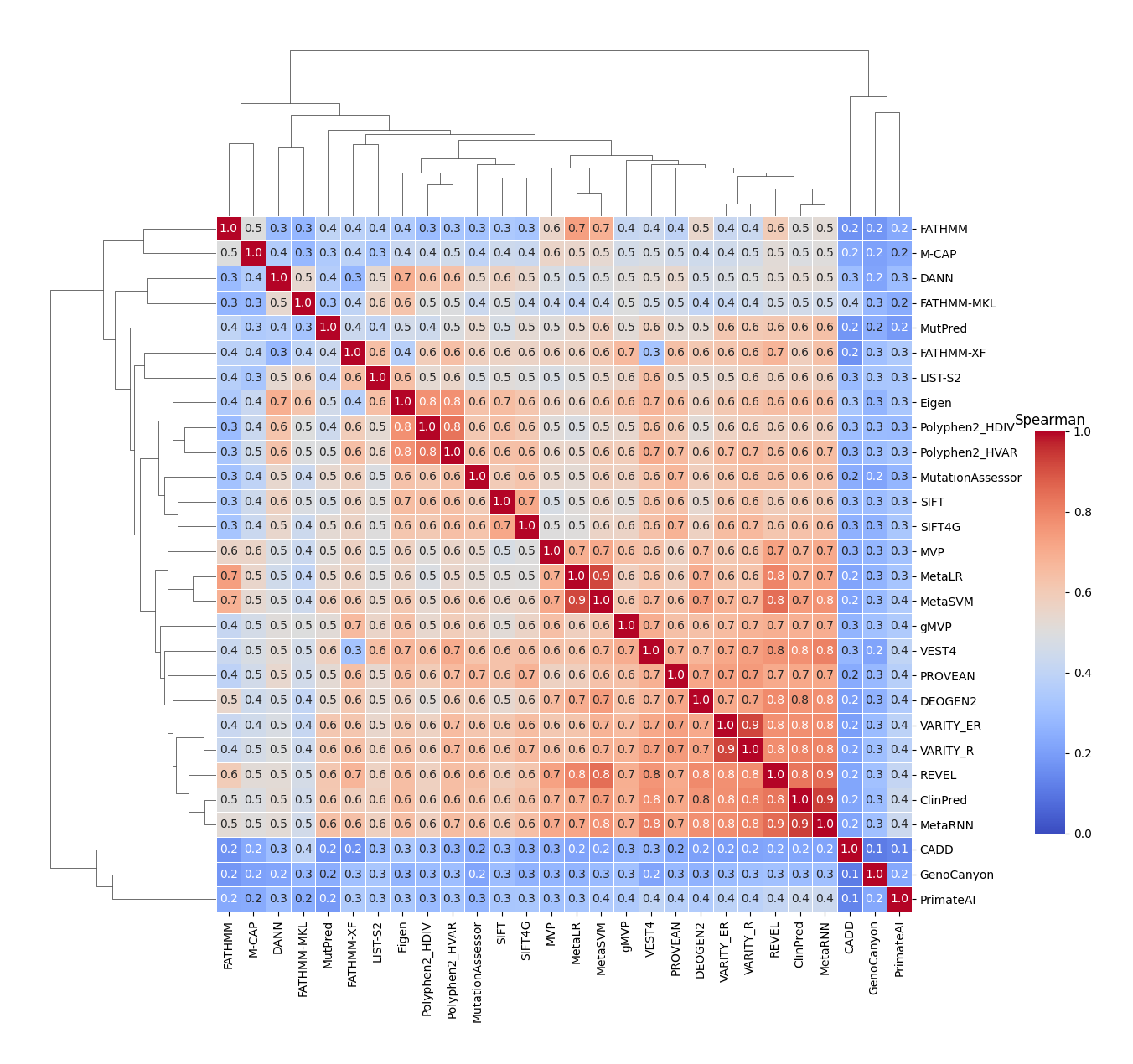


Fig. S2 Correlation of binary classification among 28 prediction methods.

The heatmap displays the Spearman rank correlation coefficients between prediction methods, and the hierarchical clustering reveals their relationships and similarities (N = 8,508).

**
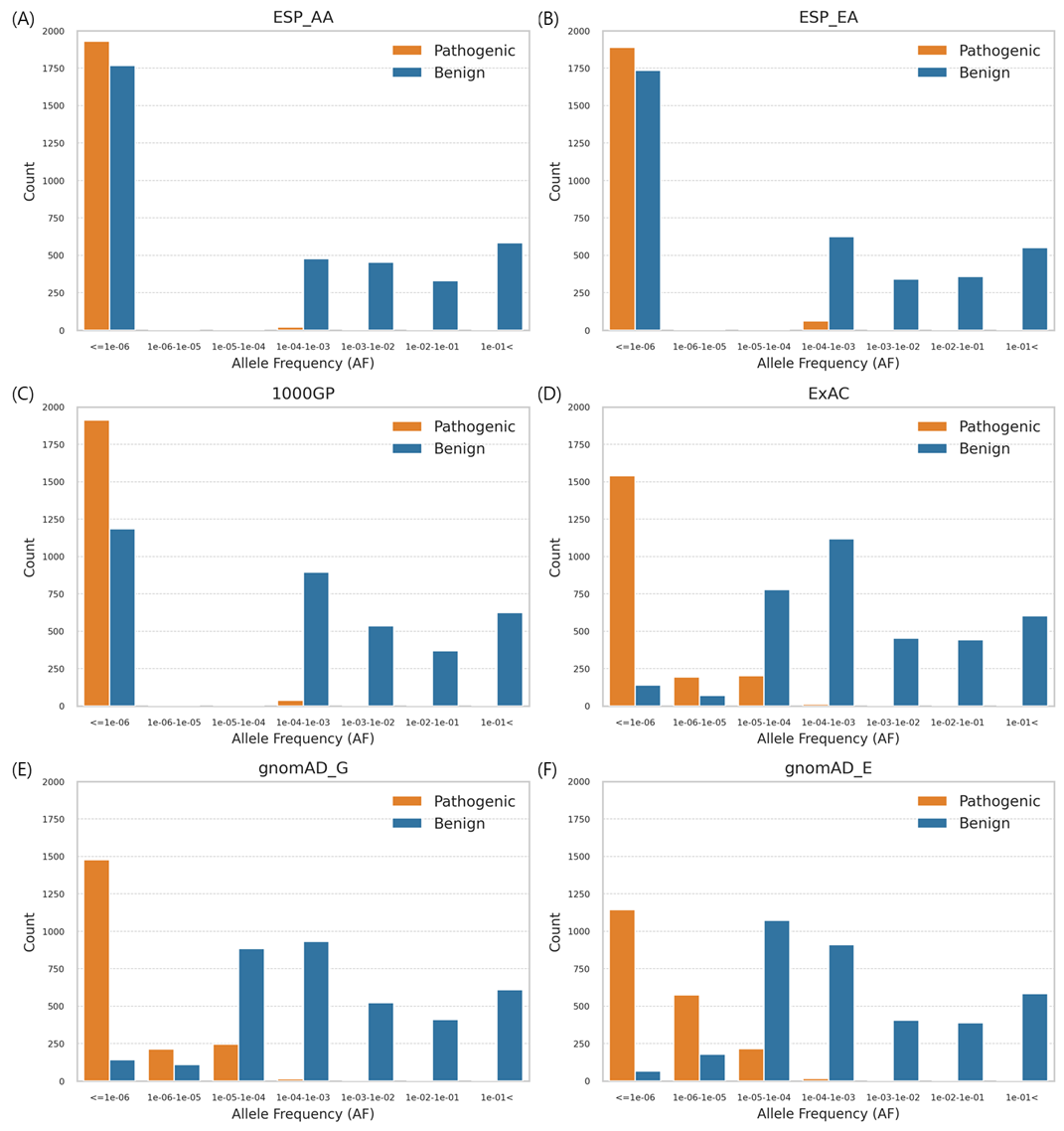
**

Fig. S3 Distribution of missense and start_lost variants across various AF ranges in six AF datasets (N=5,563).

The x-axis represents AF ranges, while the y-axis indicates the number of variants. (A) The African American samples of the Exome Sequencing Project (ESP_AA, N=2,217). (B) The European American samples of the Exome Sequencing Project (ESP_EA, N=4,298). (C) The total samples of 1000 Genomes Project (1000GP, phase3, N=2,504). (D) The total samples of the Exome Aggregation Consortium (ExAC, v0.3, N=60,706). (E) The whole genome samples of the Genome Aggregation Database (gnomAD_G, v4.0, N=76,215). (F) The whole exome samples of the Genome Aggregation Database (gnomAD_E, v4.0, N=730,947).


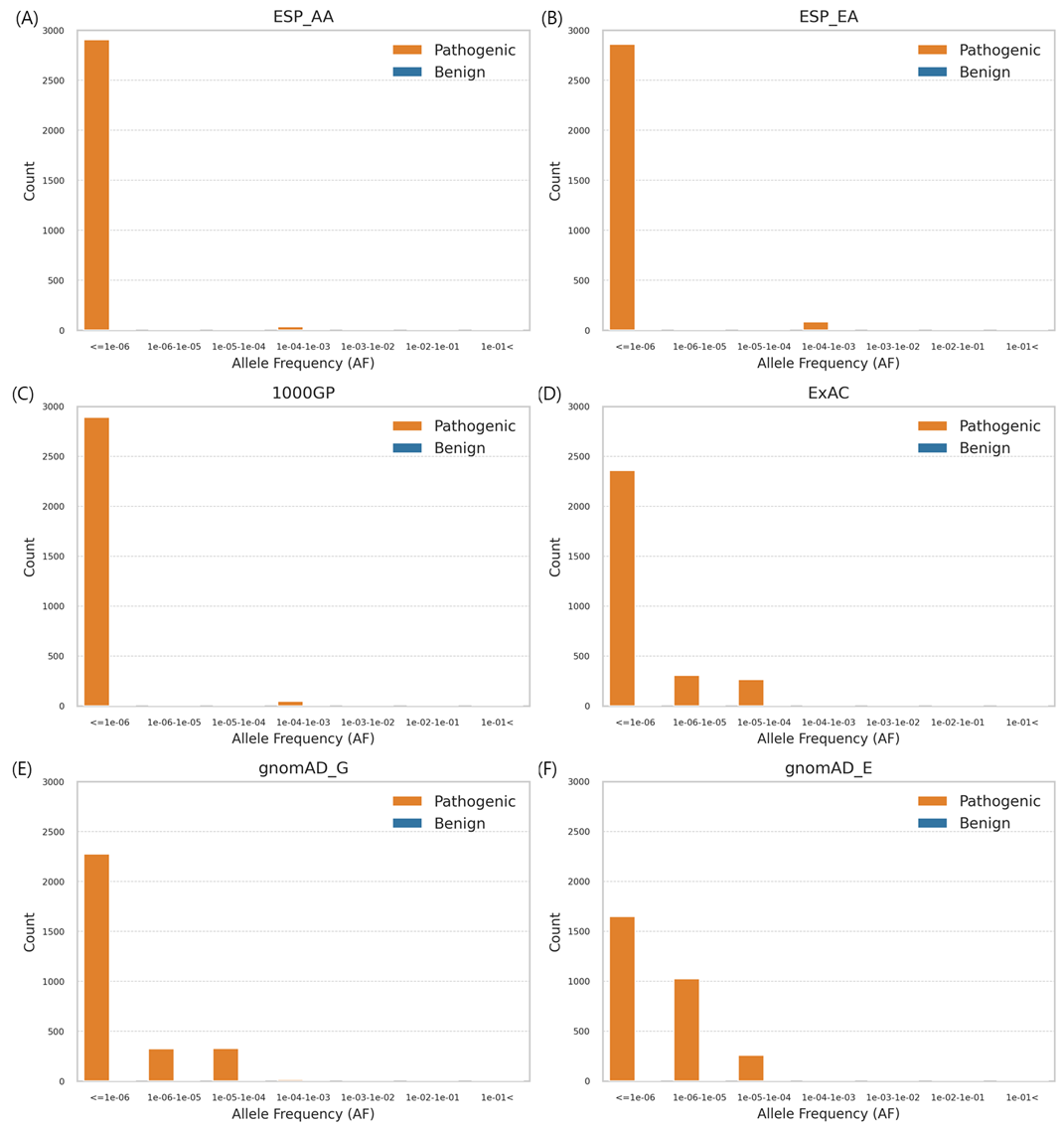


Fig. S4 Distribution of stop_gained and stop_lost variants across various AF ranges in six AF datasets (N=2,945). The x-axis represents AF ranges, while the y-axis indicates the number of variants.

**
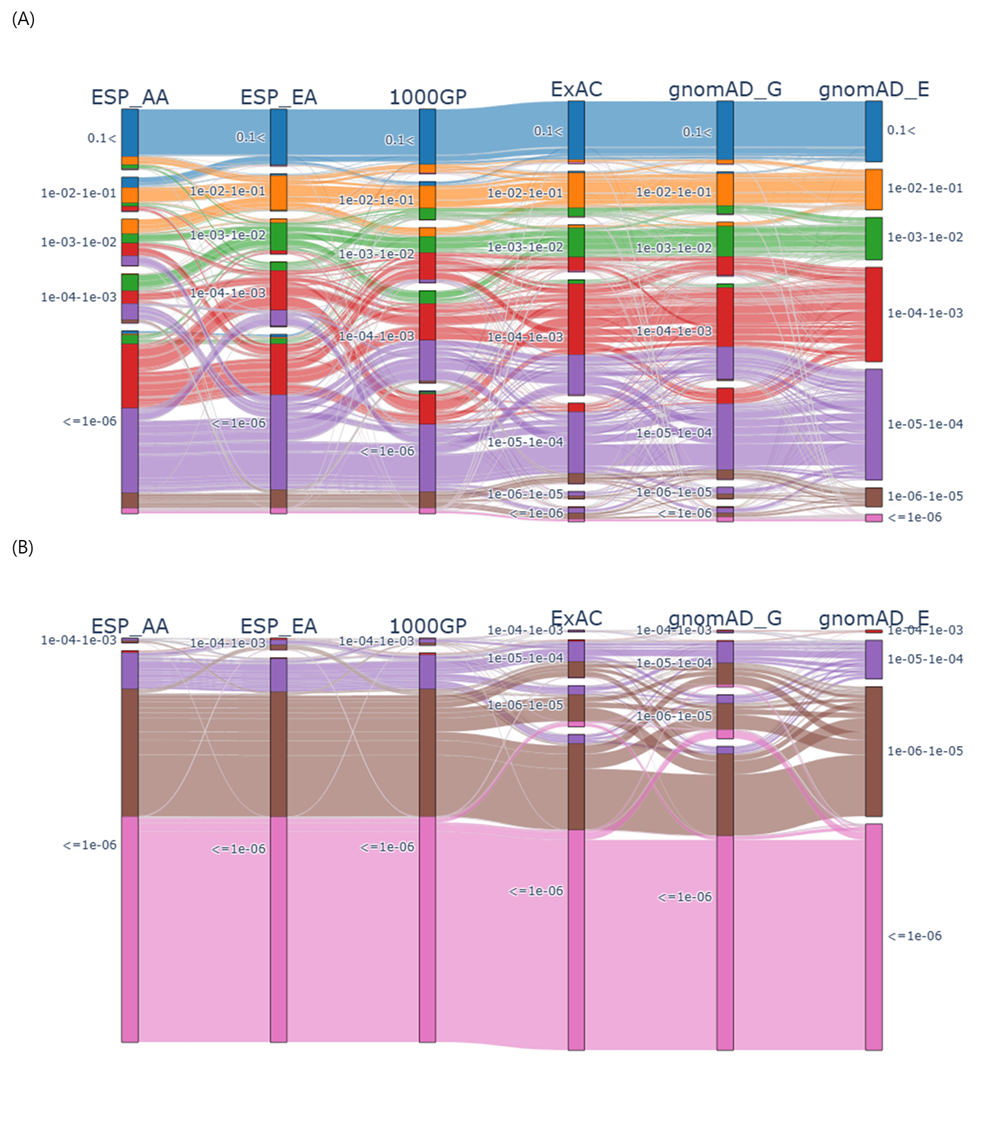
**

Fig. S5 Parallel category diagram across different AF ranges in six AF datasets (N=8,508).

Each column represents one of the six AF datasets, and each row corresponds to a variant within a specific AF range. Six colors represent AF ranges in gnomAD_E (exome). (A) Benign variants (N=3,617) (B) Pathogenic variants (N=4,891).


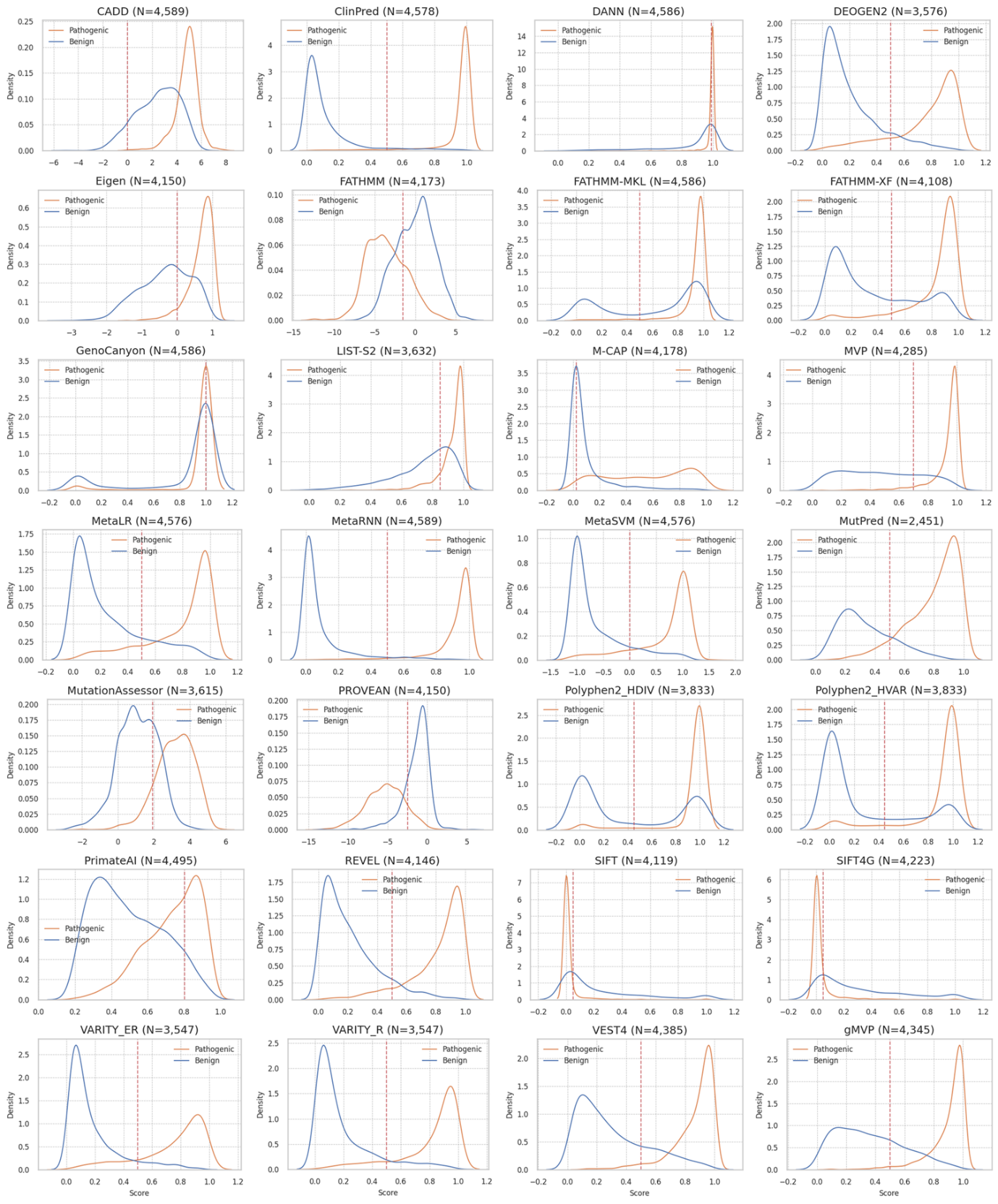


Fig. S6 Distribution of prediction scores across 28 prediction methods on rare variants.

The orange and blue lines represent pathogenic and benign variants, respectively (N=4,589). The red vertical line indicates the threshold.


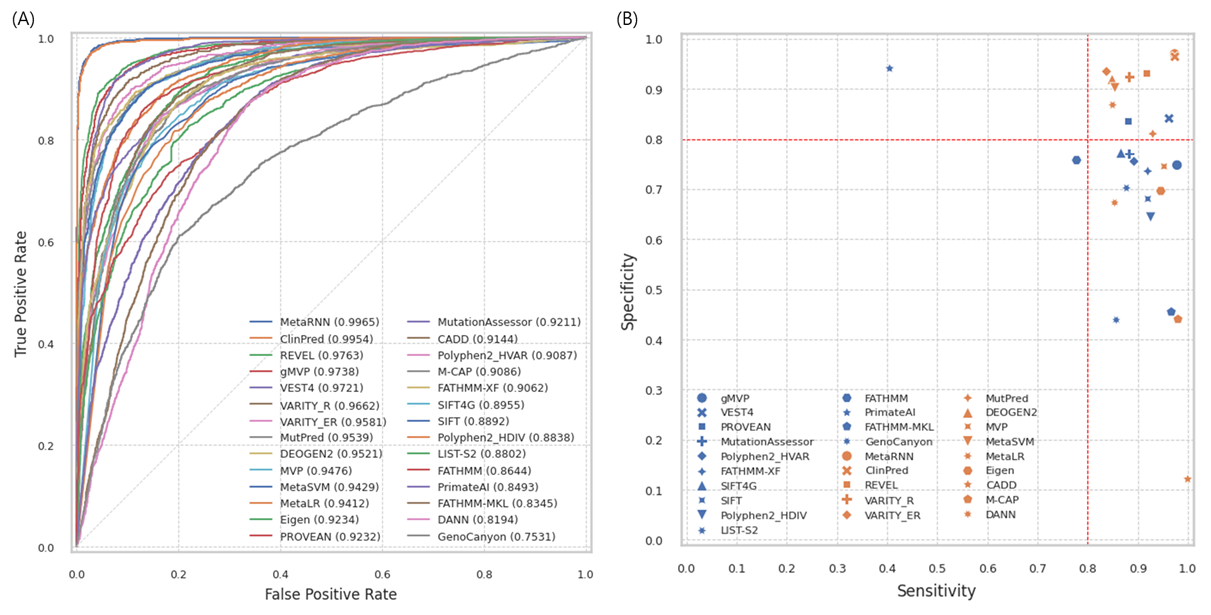


Fig. S7 Performance comparison of 28 prediction methods on all variants (N=5,563).

(A) The ROC curve shows the performance comparison of 28 prediction methods. (B) The sensitivity and specificity plot illustrates the relationship between sensitivity and specificity.


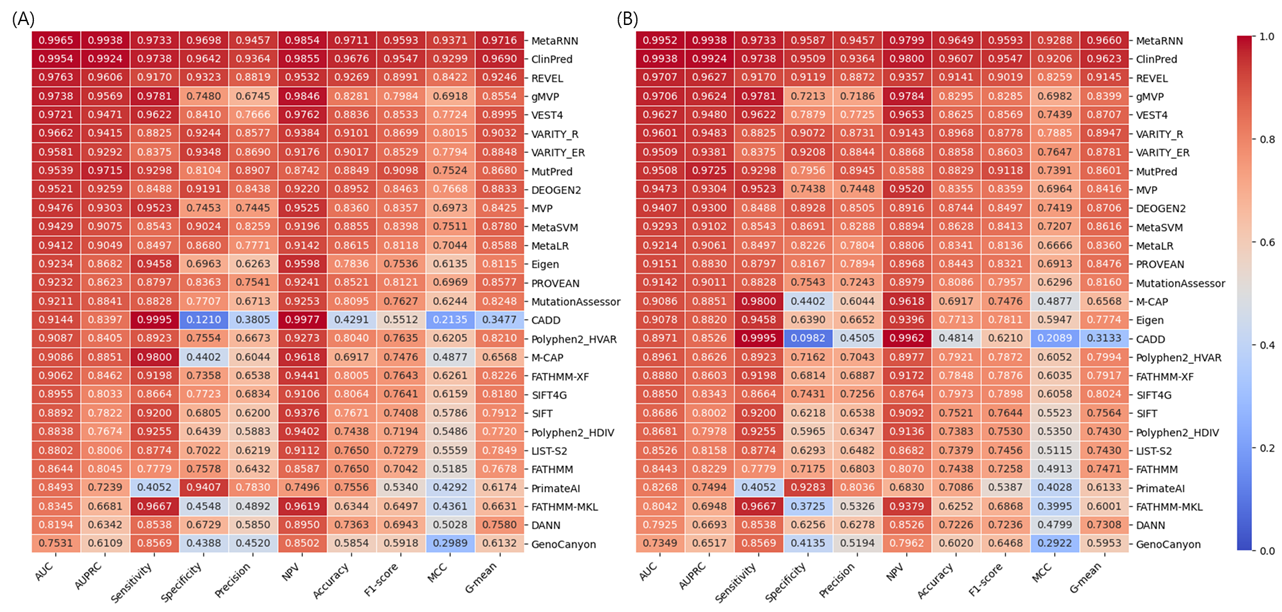


Fig. S8 Heatmap of 10 performance metrics of variants with known clinical significance.

(A) All missense and start_lost variants (N=5,563) (B) Rare missense and start_lost variants (N=4,589).


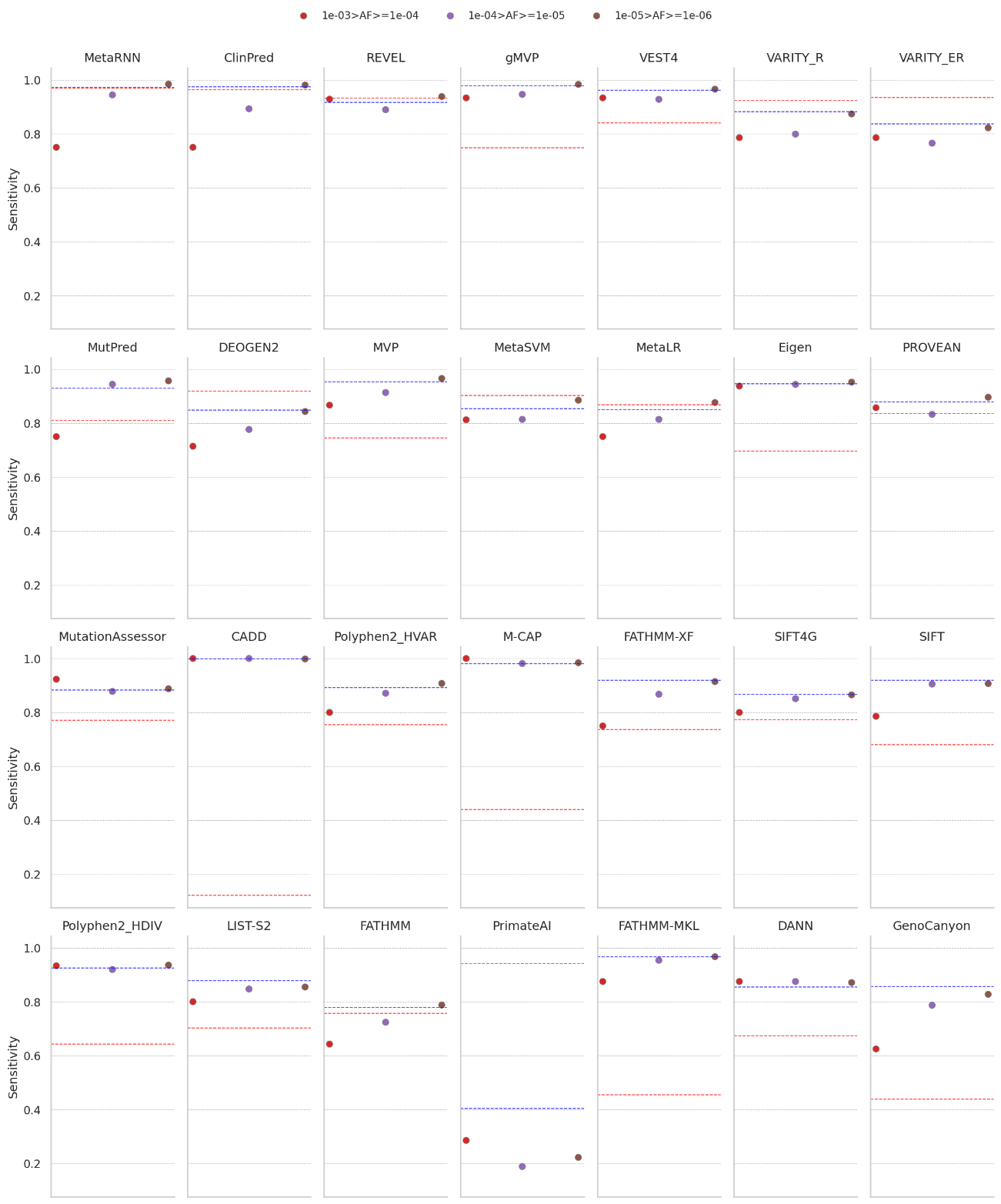


Fig. S9 Sensitivity performance of 28 prediction methods across three AF ranges.

Sensitivity tends to be higher for variants with lower AFs across the methods. The red horizontal line represents specificity, and the blue horizontal line represents sensitivity on all variants (N=5,563). The methods are ordered in descending order of AUC.
